# Supplementary material for: Assessing the effect of social contact structure on the impact of pneumococcal conjugate vaccines
Source: Sci Rep. 2025 Oct 7;15:35012. doi: 10.1038/s41598-025-20255-8 (PMC12504593; doi:10.1038/s41598-025-20255-8)
Supplement: Supplementary file 1 — Supplementary Material 1 [file 41598_2025_20255_MOESM1_ESM.docx]

**Assessing the effect of social contact structure on the impact of pneumococcal conjugate vaccines**

Anabelle Wong^1,2,*^, Sarah C. Kramer^1^, Daniel M. Weinberger^3,4^, Matthieu Domenech de Cellès^1^

1. Max Planck Institute for Infection Biology, Charitéplatz 1, 10117 Berlin, Germany.
2. Institute of Public Health, Charité – Universitätsmedizin Berlin, Charitéplatz 1, 10117 Berlin, Germany.
3. Public Health Modeling Unit, Yale School of Public Health, New Haven, CT 06511, USA.
4. Department of Epidemiology of Microbial Diseases, Yale School of Public Health, New Haven, CT 06511, USA.

* Correspondance: wong@mpiib-berlin.mpg.de

**Supplementary materials**

**Supplementary materials**

| Supplementary Figure 1 | A flowchart of the scoping literature review on the duration of carriage | 1 |
| --- | --- | --- |
| Supplementary Figure 2 | Modelled vs. observed duration of carriage with increasing age | 3 |
| Supplementary Figure 3 | Transforming the contact matrices from Mistry et al. 2021 | 4 |
| Supplementary Figure 4 | Checking model assumption: neutral null model | 7 |
| Supplementary Table 1 | Model structure: state variables | 8 |
| Supplementary Figure 5 | A flowchart of the scoping literature review on the pre-PCV prevalence of carriage | 11 |
| Supplementary Figure 6 | Assumed vs. observed carriage prevalences and different susceptibility distributions over age | 13 |
| Supplementary Figure 7 | Effect of changing vaccine efficacy and coverage on time-to-elimination | 15 |
| Supplementary Figure 8 | Effect of initial proportions of VT, NVT and Co-carriers on time-to-elimination, with changing initial proportion of VT among all colonizing serotypes ($F$) | 16 |
| Supplementary Figure 9 | Effect of initial proportions of VT, NVT and Co-carriers on time-to-elimination, with fixed initial proportion of VT among all colonizing serotypes ($F$) | 17 |
| Supplementary Figure 10 | Effect of competition on time-to-elimination with fixed proportion of VT among all colonizing serotypes ($F$) | 18 |
| Supplementary Figure 11 | Simulated time-to-elimination using type I demography vs. empirical demography | 19 |
| Supplementary Figure 12 | Contact features by age group based on type I vs. empirical demography | 20 |
| Supplementary Figure 13 | Association of features of contact patterns and time-to-elimination simulated with type I demography in all age groups | 21 |
| Supplementary Figure 14 | Association of features of contact patterns and time-to-elimination simulated with empirical demography in all age groups | 23 |
| Supplementary Figure 15 | Association of features of contact patterns and time-to-elimination assuming different carriage prevalences | 24 |
| Supplementary Table 2 | Out-of-sample prediction using contact rate and assortativity as predictors | 25 |
| References | | 26 |
| Supplementary Data 1 | Extracted data for fitting clearance rate  (available as a data file) |  |
| Supplementary Data 2 | Extracted data for checking assumption on carriage prevalence  (available as a data file) |  |
| Supplementary Data 3 | Extracted data for verifying simulated VT-carriage in children  (available as a data file) |  |

**Supplementary Figure 1. A flowchart of the scoping literature review on the duration of carriage**

We searched for observational studies on pneumococcal carriage duration on PubMed and identified additional relevant studies from the references of the initially included studies. We selected culture-based studies to allow the inclusion of the maximum number of studies because the majority of the early studies relied on culture-based detection. Further, because the duration of carriage has a left-skewed distribution, with few individuals showing lasting carriage, we preferred median to mean reporting and included studies that reported median duration. A total of 8 studies were included [1–8].

**Supplementary Figure 2. Modelled vs. observed duration of carriage with increasing age**


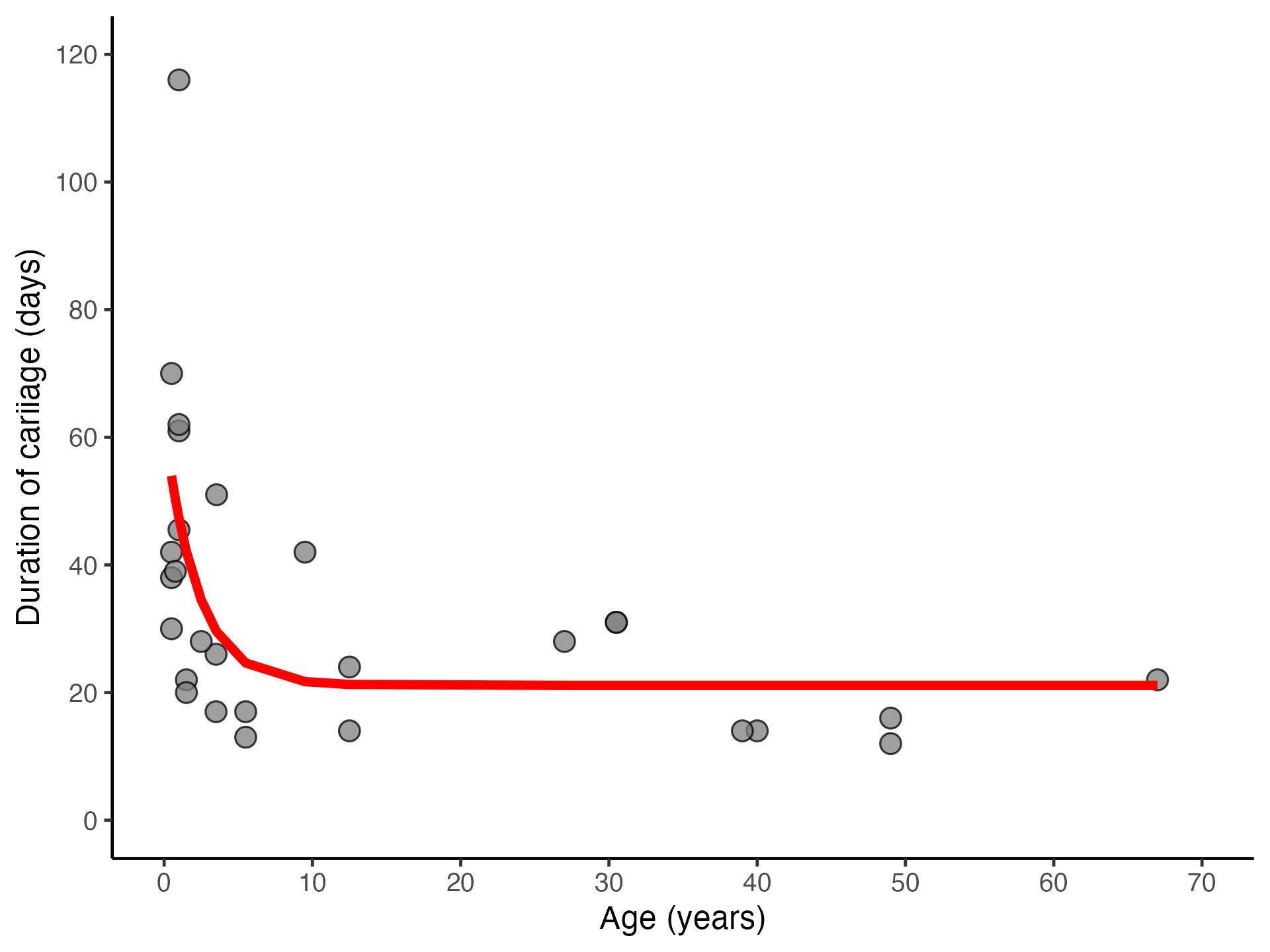


To obtain the duration of carriage, we fitted a function of age to the extracted duration of carriage from published longitudinal carriage studies identified through a scoping literature search (Data 1). For the model of carriage duration with age, we used the non-linear least square algorithm to estimate the parameters in the equation:

$$Duration=a+(b-a)\times exp(-c\times Age)$$

where $a=21$ (standard error: 5.8), $b=62 (14.6)$, and $c=0.45 (0.4)$.

Supplementary Figure 2 shows the extracted data from observational studies as grey points and the modeled duration of carriage with a red curve.

**Supplementary Figure 3. Transforming the contact matrices from Mistry et al. 2021**

**
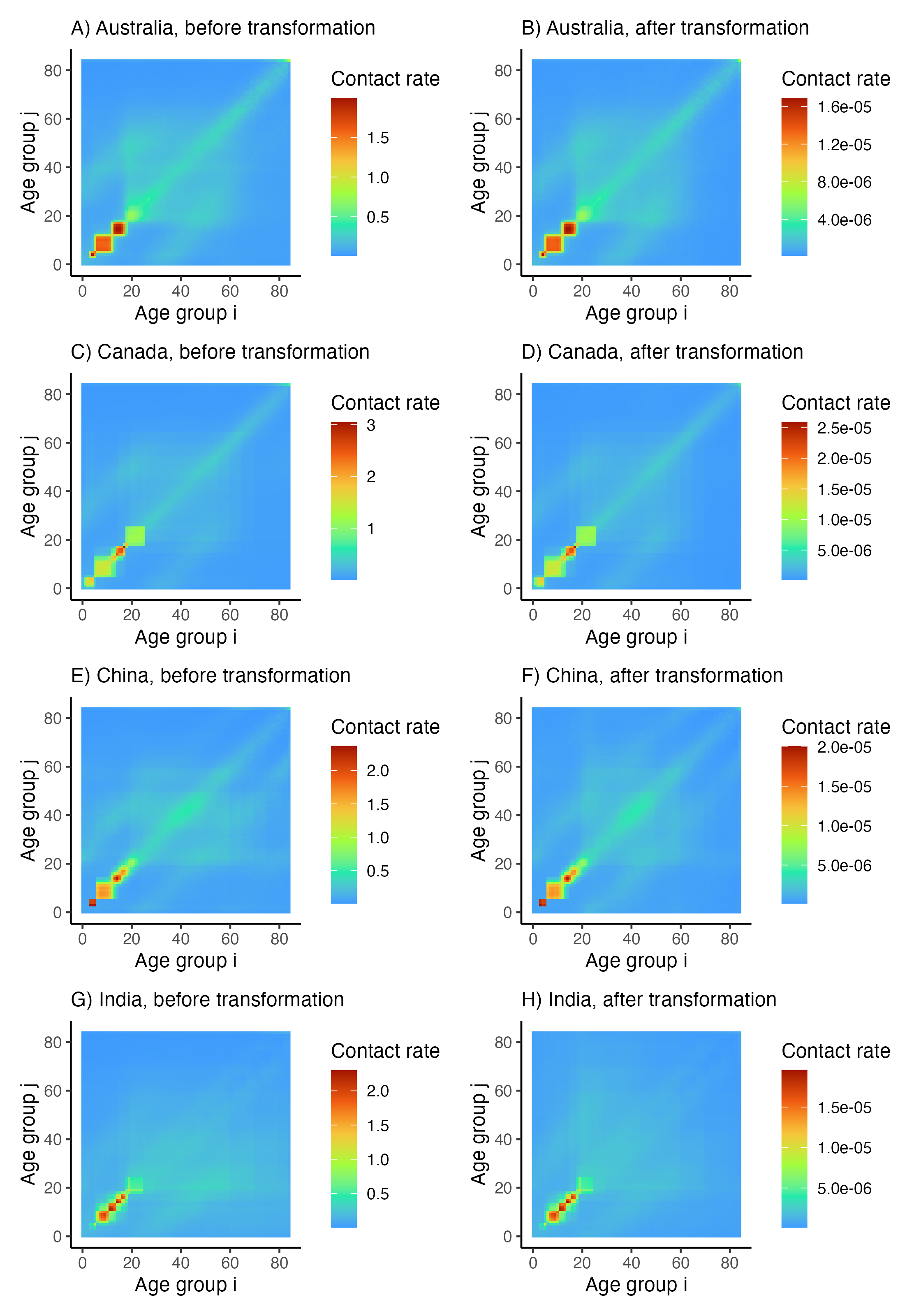
**


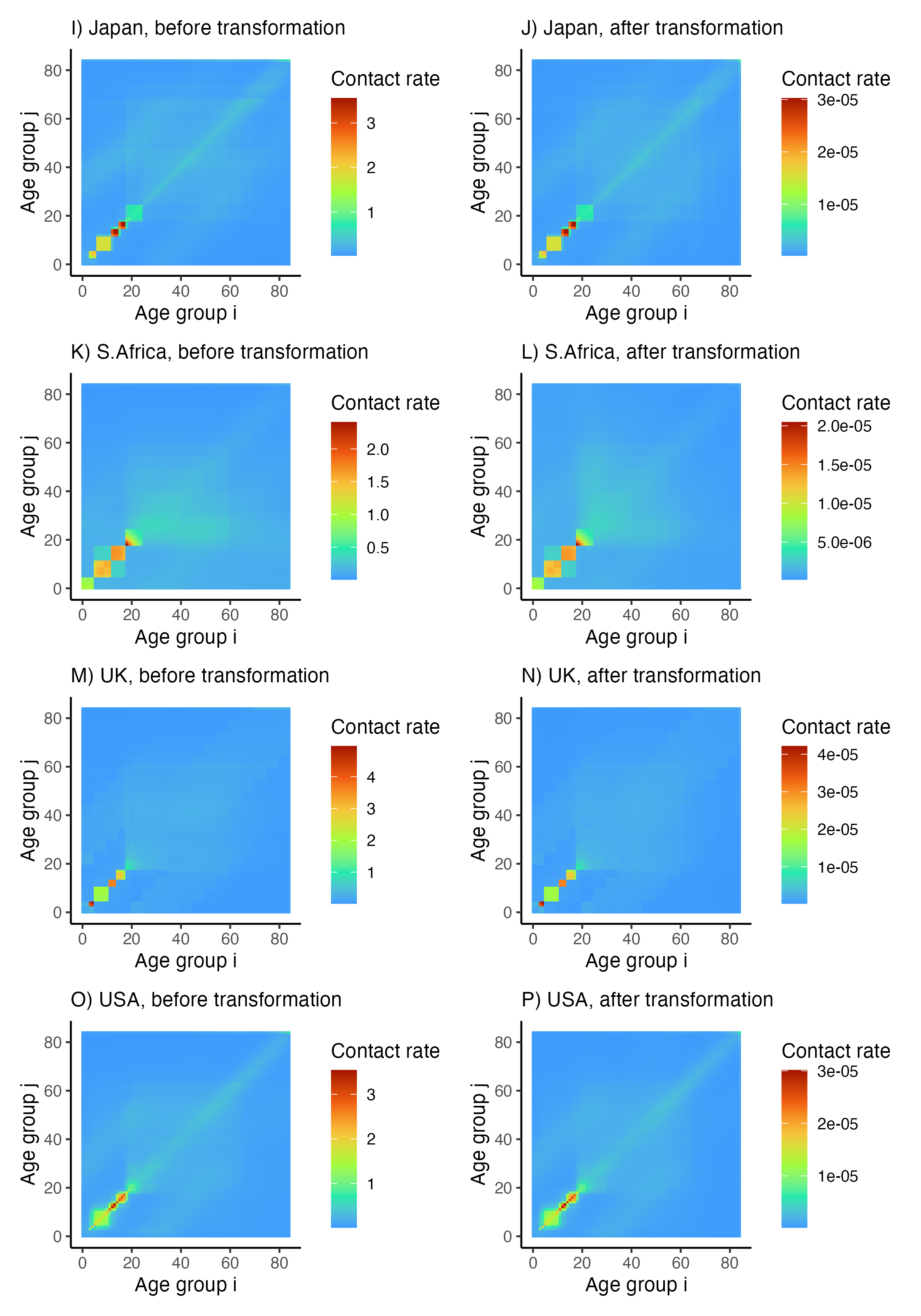


Let ${M=(M}_{ij})$ represent the original contact rate matrix calculated in [9]. By definition, $M_{ij}= \frac{E_{ij}}{N_{i}}$ , where $E_{ij}$ represents the number of daily contacts between age groups $i$ and $j$, and $N_{i}$ the population size of age group $i$. Because of the necessary reciprocity of total contacts (i.e., $E_{ij}$ = $E_{ji}$), the per capita contact matrix $m_{ij}=\frac{M_{ij}}{N_{j}}=\frac{E_{ij}}{N_{i}N_{j}}$ should be symmetric. To ensure this symmetry, one can calculate a contact matrix $\tilde{M}$ corrected for reciprocity based on the population structure in the study population [10]:

$$\tilde{M}_{ij}= \frac{1}{2N_{i}}(M_{ij}N_{i}+M_{ji}N_{j})$$

As a result, the per capita contact matrix:

$$\tilde{m}_{ij}= \frac{M_{ij}N_{i}+M_{ji}N_{j}}{2N_{i}N_{j}}$$

is symmetric, as it should be.

Finally, we multiplied the per capita matrix by 365 to obtain a per capita annual contact matrix because all rates were per year in the simulations.

Supplementary Figure 3 (A–P) shows the pre- and post-transformation contact patterns of a subset of 8 out of the 34 contact matrices as examples.

**Supplementary Figure 4. Checking model assumption: neutral null model**


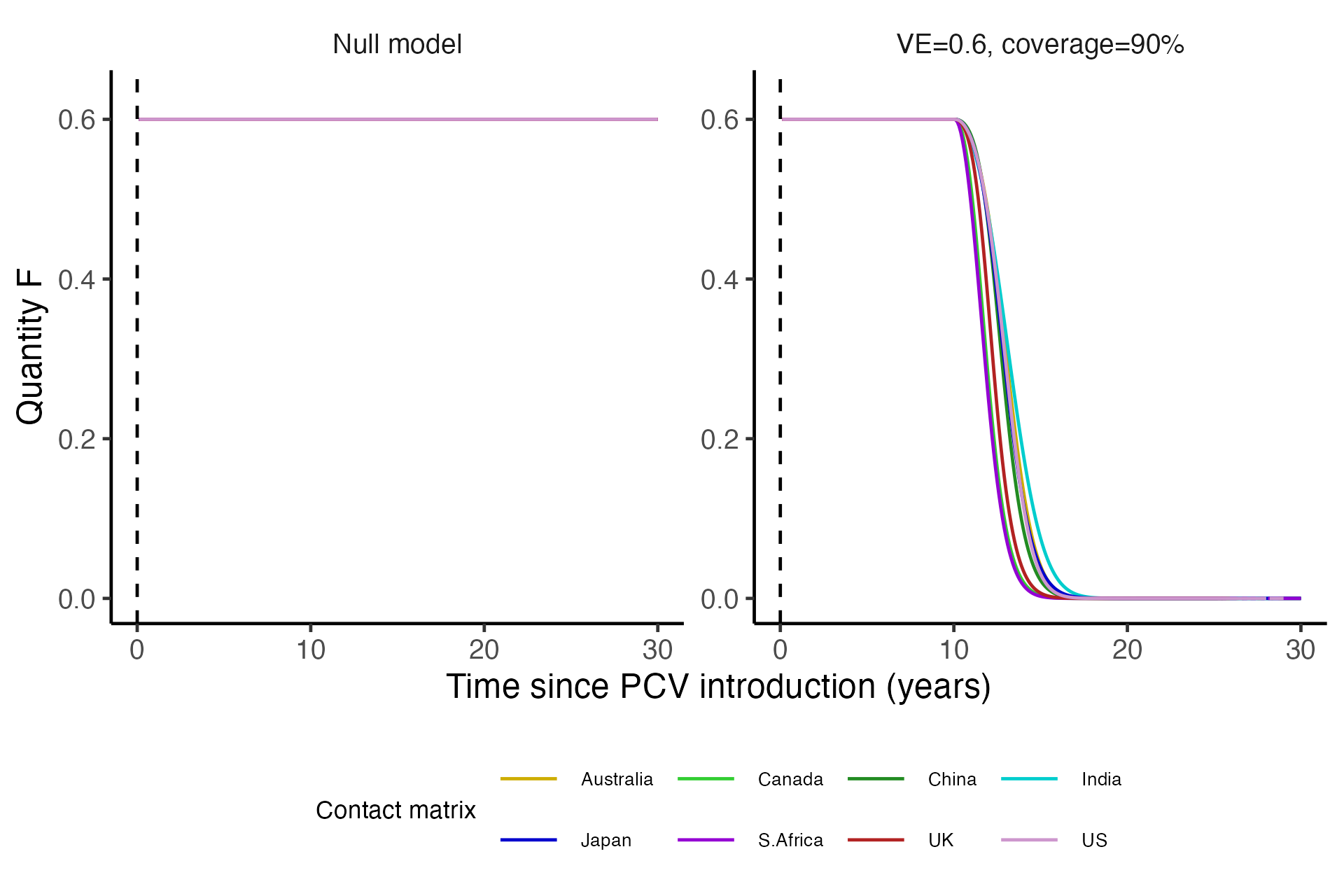


To check if our null model fulfils the neutrality criterion, we tracked proportion of VT among all colonizing serotypes ($F$) as proposed by [11]. The quantity $F$ is given by:

$$F= \frac{C_{V}^{(V)}+C_{V}^{(N)}+q(C_{VN}^{\left( V \right)}+C_{VN}^{(N)})}{C_{V}^{(V)}+C_{V}^{(N)}{+ C}_{N}^{(V)}+C_{N}^{(N)}+2q(C_{VN}^{\left( V \right)}+C_{VN}^{(N)})}$$

In a neutral null model, where one serotype is not assumed to have a fitness advantage over the other, any level of co-existence should be permitted and the proportions of the two serotypes should not converge to a global equilibrium (e.g., $F$ = 50%) without explicit mechanism [11]. In other words, neutral models do not lead to a 50-50 coexistence by default. Specifically, $F$ should remain constant regardless of the initial proportions of VT and NVT under no intervention, where the intervention represents a mechanism that gives one serotype a fitness advantage over the other.

The left panel of Supplementary Figure 4 shows that *F* remained stable when a null-impact vaccine was introduced for all contact matrices. The right panel shows that *F* declined after the introduction of a vaccine with vaccine efficacy=0.6 at 90% coverage, at different rates in different contact structures.

**Supplementary Table 1. Model structure: state variables**

| **State variable** | **Definition** |
| --- | --- |
| $S^{(V,i)}$ | Susceptible, vaccinated, age group $i$ |
| $C_{V}^{(V,i)}$ | Colonized by VT, vaccinated, age group $i$ |
| $C_{N}^{(V,i)}$ | Colonized by NVT, vaccinated, age group $i$ |
| $C_{VN}^{(V,i)}$ | Colonized by VT and NVT, vaccinated, age group $i$ |
| $S^{(N,i)}$ | Susceptible, unvaccinated, age group $i$ |
| $C_{V}^{(N,i)}$ | Colonized by VT, unvaccinated, age group $i$ |
| $C_{N}^{(N,i)}$ | Colonized by NVT, unvaccinated, age group $i$ |
| $C_{VN}^{(V,i)}$ | Colonized by VT and NVT, unvaccinated, age group $i$ |

The transmission dynamic is described by the following system of ordinary differential equations:

**Equations in (unvaccinated) newborns (**$i=0$**)**

Newborns are assumed to be non-carriers and, therefore, directly enter the $S^{(N,0)}$ compartment.

$\delta_{0}$ represents the aging rate, equal to $\frac{1}{a_{0}}$, where $a_{0}$ represents the age span of age group 0.

$N=\sum_{i=0}^{A-1} N_{i}$ is the total population size (summed across all age groups).

$\mu$ is the per capita birth rate. To keep the population constant, this is calculated as $\mu^{-1}=a=\sum_{i} a_{i}$, where $a_{i}$ is the age span of age groups $i$ and $a$ represents the assumed lifespan. For example, if one assumes a lifespan $a=80$ years, then the birth rate equals $\mu=a^{-1}=\frac{1}{80}$ per year.

In the main analysis, newborns are born at the rate $\mu N$, then individuals age across the age groups, and all die exactly at the age of 84. This simplified demographic model is known as type-I mortality distribution [12].

$$\frac{dS^{(V,0)}}{dt}=0$$

$$\frac{dC_{V}^{(V,0)}}{dt}=0$$

$$\frac{dC_{N}^{(V,0)}}{dt}=0$$

$$\frac{dC_{VN}^{(V,0)}}{dt}=0$$

$$\frac{dS^{(N,0)}}{dt}= \mu N-(\lambda_{V}^{\left( 0 \right)}+\lambda_{N}^{\left( 0 \right)})S^{\left( N,0 \right)} +\gamma_{0}(C_{V}^{\left( N,0 \right)}+C_{N}^{\left( N,0 \right)}+ C_{VN}^{\left( N,0 \right)})-\delta_{0}S^{(N,0)}$$

$$\frac{dC_{V}^{(N,0)}}{dt}=\lambda_{V}^{\left( 0 \right)}{(S}^{(N,0)}+c k_{V}C_{VN}^{(N,0)}) -k_{N}\lambda_{N}^{\left( 0 \right)}C_{V}^{\left( N,0 \right)}-{(\gamma}_{0}+\delta_{0})C_{V}^{(N,0)}$$

$$\frac{dC_{N}^{\left( N,0 \right)}}{dt}=\lambda_{N}^{\left( 0 \right)}{(S}^{(N,0)}+c k_{N}C_{VN}^{(N,0)}) -k_{V}\lambda_{V}^{\left( 0 \right)}C_{N}^{\left( N,0 \right)}-{(\gamma}_{0}+\delta_{0})C_{N}^{(N,0)}$$

$$\frac{dC_{VN}^{(N,0)}}{dt}=k_{N}\lambda_{N}^{\left( 0 \right)}C_{V}^{(N,0)}+k_{V}\lambda_{V}^{\left( 0 \right)}C_{N}^{(N,0)}-c(k_{N}\lambda_{N}^{\left( 0 \right)}+k_{V}\lambda_{V}^{\left( 0 \right)}{)C}_{VN}^{(N,0)}-{(\gamma}_{0}+\delta_{0})C_{VN}^{(N,0)}$$

**Equations in infants receiving vaccination (**$i=1$**)**

Write $v\left( t \right)=p_{V} for (t\geq t_{V} )$, the time-varying vaccine coverage, where $t_{V}$ represents the time point of PCV introduction and $pV$ the proportion of infants vaccinated after PCV introduction.

Vaccination is assumed to occur at age 0, that is, when the newborns age to the second age group. The other properties of the vaccine are its efficacy against acquisition of V-serotypes (denoted by $\epsilon_{V}$) and its rate of waning protection (denoted by $\alpha_{V}$, where $1/\alpha_{V}$ represents the average duration of vaccine protection).

$$\frac{dS^{(V,1)}}{dt}= v\left( t \right)\delta_{0}S^{(N,0)}-[\lambda_{V}^{\left( 1 \right)}(1-\epsilon_{V})+\lambda_{N}^{\left( 1 \right)}]S^{\left( V,1 \right)} +\gamma_{1}(C_{V}^{\left( V,1 \right)}+C_{N}^{\left( V,1 \right)}+ C_{VN}^{\left( V,1 \right)})-({\alpha_{V}+\delta}_{1})S^{(V,1)}$$

$$\frac{dC_{V}^{(V,1)}}{dt}={v\left( t \right)\delta_{0}C_{V}^{(N,0)}+ \lambda}_{V}^{\left( 1 \right)}{(1-\epsilon_{V})(S}^{(V,1)}+c k_{V}C_{VN}^{(V,1)}) -k_{N}\lambda_{N}^{\left( 1 \right)}C_{V}^{\left( V,1 \right)}-{(\alpha_{V}+\gamma}_{1}+\delta_{1})C_{V}^{(V,1)}$$

$$\frac{dC_{N}^{\left( V,1 \right)}}{dt}={v\left( t \right)\delta_{0}C_{N}^{(N,0)}+\lambda}_{N}^{\left( 1 \right)}{(S}^{(V,1)}+c k_{N}C_{VN}^{(V,1)}) -k_{V}\lambda_{V}^{\left( 1 \right)}{(1-\epsilon_{V})C}_{N}^{\left( V,1 \right)}-{(\alpha_{V}+\gamma}_{1}+\delta_{1})C_{N}^{(V,1)}$$

$$\frac{dC_{VN}^{(V,1)}}{dt}={v\left( t \right)\delta_{0}C_{VN}^{(N,0)}+k}_{N}\lambda_{N}^{\left( 1 \right)}C_{V}^{(V,1)}+k_{V}\lambda_{V}^{\left( 1 \right)}(1-\epsilon_{V})C_{N}^{\left( V,1 \right)}-c[k_{N}\lambda_{N}^{\left( 1 \right)}+k_{V}\lambda_{V}^{\left( 1 \right)}(1-\epsilon_{V}){]C}_{VN}^{(V,1)}-{(\alpha_{V}+\gamma}_{1}+\delta_{1})C_{VN}^{(V,1)}$$

$$\frac{dS^{(N,1)}}{dt}= \left[ 1-v\left( t \right) \right]\delta_{0}S^{\left( N,0 \right)}-{(\lambda}_{V}^{\left( 1 \right)}+\lambda_{N}^{\left( 1 \right)})S^{\left( N,1 \right)} +\gamma_{1}\left( C_{V}^{\left( N,1 \right)}+C_{N}^{\left( N,1 \right)}+ C_{VN}^{\left( N,1 \right)} \right)+{\alpha_{V}S^{(V,1)}-\delta}_{1}S^{(N,1)}$$

$$\frac{dC_{V}^{(N,1)}}{dt}={\left[ 1-v\left( t \right) \right]\delta_{0}C_{V}^{(N,0)}+ \lambda}_{V}^{\left( 1 \right)}{(S}^{(N,1)}+c k_{V}C_{VN}^{(N,1)}) -k_{N}\lambda_{N}^{\left( 1 \right)}C_{V}^{\left( N,1 \right)}+{\alpha_{V}C_{V}^{(V,1)}-(\gamma}_{1}+\delta_{1})C_{V}^{(N,1)}$$

$$\frac{dC_{N}^{\left( N,1 \right)}}{dt}={\left[ 1-v\left( t \right) \right]\delta_{0}C_{N}^{(N,0)}+\lambda}_{N}^{\left( 1 \right)}{(S}^{(N,1)}+c k_{N}C_{VN}^{(N,1)}) -k_{V}\lambda_{V}^{\left( 1 \right)}C_{N}^{\left( N,1 \right)}+{\alpha_{V}C_{N}^{(V,1)}-(\gamma}_{1}+\delta_{1})C_{N}^{(N,1)}$$

$$\frac{dC_{VN}^{(N,1)}}{dt}={\left[ 1-v\left( t \right) \right]\delta_{0}C_{VN}^{(N,0)}+k}_{N}\lambda_{N}^{\left( 1 \right)}C_{V}^{(N,1)}+k_{V}\lambda_{V}^{\left( 1 \right)}C_{N}^{\left( N,1 \right)}-c(k_{N}\lambda_{N}^{\left( 1 \right)}+k_{V}\lambda_{V}^{\left( 1 \right)})C_{VN}^{(N,1)}+{\alpha_{V}C_{VN}^{(V,1)}-(\gamma}_{1}+\delta_{1})C_{VN}^{(N,1)}$$

**Equations in older age groups (**$i=2,\ldots, A-1$**)**

In the older age groups, no more vaccination is assumed, and the dynamic is described by the following system of ordinary differential equations:

$$\frac{dS^{(V,i)}}{dt}= \delta_{i-1}S^{(V,i-1)}-[\lambda_{V}^{\left( i \right)}(1-\epsilon_{V})+\lambda_{N}^{\left( i \right)}]S^{\left( V,i \right)} +\gamma_{i}(C_{V}^{\left( V,i \right)}+C_{N}^{\left( V,i \right)}+ C_{VN}^{\left( V,i \right)})-({\alpha_{V}+\delta}_{i})S^{(V,i)}$$

$$\frac{dC_{V}^{(V,i)}}{dt}={\delta_{i-1}C_{V}^{(V,i-1)}+ \lambda}_{V}^{\left( i \right)}{(1-\epsilon_{V})(S}^{(V,i)}+c k_{V}C_{VN}^{(V,i)}) -k_{N}\lambda_{N}^{\left( i \right)}C_{V}^{\left( V,i \right)}-{(\alpha_{V}+\gamma}_{i}+\delta_{i})C_{V}^{(V,i)}$$

$$\frac{dC_{N}^{\left( V,i \right)}}{dt}={\delta_{i-1}C_{N}^{(V,i-1)}+\lambda}_{N}^{\left( i \right)}{(S}^{(V,i)}+c k_{N}C_{VN}^{(V,i)}) -k_{V}\lambda_{V}^{\left( i \right)}{(1-\epsilon_{V})C}_{N}^{\left( V,i \right)}-{(\alpha_{V}+\gamma}_{i}+\delta_{i})C_{N}^{(V,i)}$$

$$\frac{dC_{VN}^{(V,i)}}{dt}={\delta_{i-1}C_{VN}^{(V,i-1)}+k}_{N}\lambda_{N}^{\left( i \right)}C_{V}^{(V,i)}+k_{V}\lambda_{V}^{\left( i \right)}(1-\epsilon_{V})C_{N}^{\left( V,i \right)}-c[k_{N}\lambda_{N}^{\left( i \right)}+k_{V}\lambda_{V}^{\left( i \right)}(1-\epsilon_{V}){]C}_{VN}^{(V,i)}-{(\alpha_{V}+\gamma}_{i}+\delta_{i})C_{VN}^{(V,i)}$$

$$\frac{dS^{(N,i)}}{dt}= \delta_{i-1}S^{\left( N,i-1 \right)}-{(\lambda}_{V}^{\left( i \right)}+\lambda_{N}^{\left( i \right)})S^{\left( N,i \right)} +\gamma_{i}\left( C_{V}^{\left( N,i \right)}+C_{N}^{\left( N,i \right)}+ C_{VN}^{\left( N,i \right)} \right)+{\alpha_{V}S^{(V,i)}-\delta}_{i}S^{(N,i)}$$

$$\frac{dC_{V}^{(N,i)}}{dt}={\delta_{i-1}C_{V}^{(N,i-1)}+ \lambda}_{V}^{\left( i \right)}{(S}^{(N,i)}+c k_{V}C_{VN}^{(N,i)}) -k_{N}\lambda_{N}^{\left( i \right)}C_{V}^{\left( N,i \right)}+{\alpha_{V}C_{V}^{(V,i)}-(\gamma}_{i}+\delta_{i})C_{V}^{(N,i)}$$

$$\frac{dC_{N}^{\left( N,i \right)}}{dt}={\delta_{i-1}C_{N}^{(N,i-1)}+\lambda}_{N}^{\left( i \right)}{(S}^{(N,i)}+c k_{N}C_{VN}^{(N,i)}) -k_{V}\lambda_{V}^{\left( i \right)}C_{N}^{\left( N,i \right)}+{\alpha_{V}C_{N}^{(V,i)}-(\gamma}_{i}+\delta_{i})C_{N}^{(N,i)}$$

$$\frac{dC_{VN}^{(N,i)}}{dt}={\delta_{i-1}C_{VN}^{(N,i-1)}+k}_{N}\lambda_{N}^{\left( i \right)}C_{V}^{(N,i)}+k_{V}\lambda_{V}^{\left( i \right)}C_{N}^{\left( N,i \right)}-c(k_{N}\lambda_{N}^{\left( i \right)}+k_{V}\lambda_{V}^{\left( i \right)})C_{VN}^{(N,i)}+{\alpha_{V}C_{VN}^{(V,i)}-(\gamma}_{i}+\delta_{i})C_{VN}^{(N,i)}$$

**Initial conditions**

The model was first run to simulate the pre-vaccine era, so all corresponding initial conditions were set to $0=(X^{\left( V,i \right)}=0)$. For the initial conditions in the non-vaccinated groups, we defined the following parameters:

$f_{C}^{\left( i \right)}(0)$ : initial prevalence of carriers in age group $i$

$f_{V}\left( 0 \right)$ : proportion of VT carriers / all carriers

$f_{N}\left( 0 \right)$ : proportion of NVT carriers / all carriers

$f_{VN}\left( 0 \right)=1-f_{V}\left( 0 \right)-f_{N}\left( 0 \right)$ : proportion of dual carriers/all carriers

With these parameters, the state variables were initialized as follows:

$$S^{\left( N,i \right)}\left( 0 \right)= N_{i} \times[1-f_{C}^{\left( i \right)}(0)]$$

$$C_{V}^{(N,i)}\left( 0 \right)= N_{i} \times f_{C}^{\left( i \right)}(0)\times f_{V}\left( 0 \right)$$

$$C_{N}^{(N,i)}\left( 0 \right)= N_{i} \times f_{C}^{\left( i \right)}(0)\times f_{N}\left( 0 \right)$$

$$C_{VN}^{(N,i)}\left( 0 \right)= N_{i} \times f_{C}^{\left( i \right)}\left( 0 \right)\times[1-f_{V}\left( 0 \right)-f_{N}\left( 0 \right)]$$

**Supplementary Figure 5. A flowchart of the scoping literature review on the pre-PCV prevalence of carriage**

We searched for observational studies on pneumococcal carriage prevalence on PubMed and identified additional relevant studies from the references of the initially identified studies. We selected culture-based studies to allow the inclusion of the maximum number of studies because the majority of the early studies relied on culture-based detection. Further, we excluded studies that focused on a specific patient group, studies that report observations from the same sample included in another study (de-duplication), and studies that report only post-PCV prevalence of carriage. A total of 17 studies were included [5–8,13–25].

**Supplementary Figure 6. Assumed vs. observed carriage prevalences and different susceptibility distributions over age**


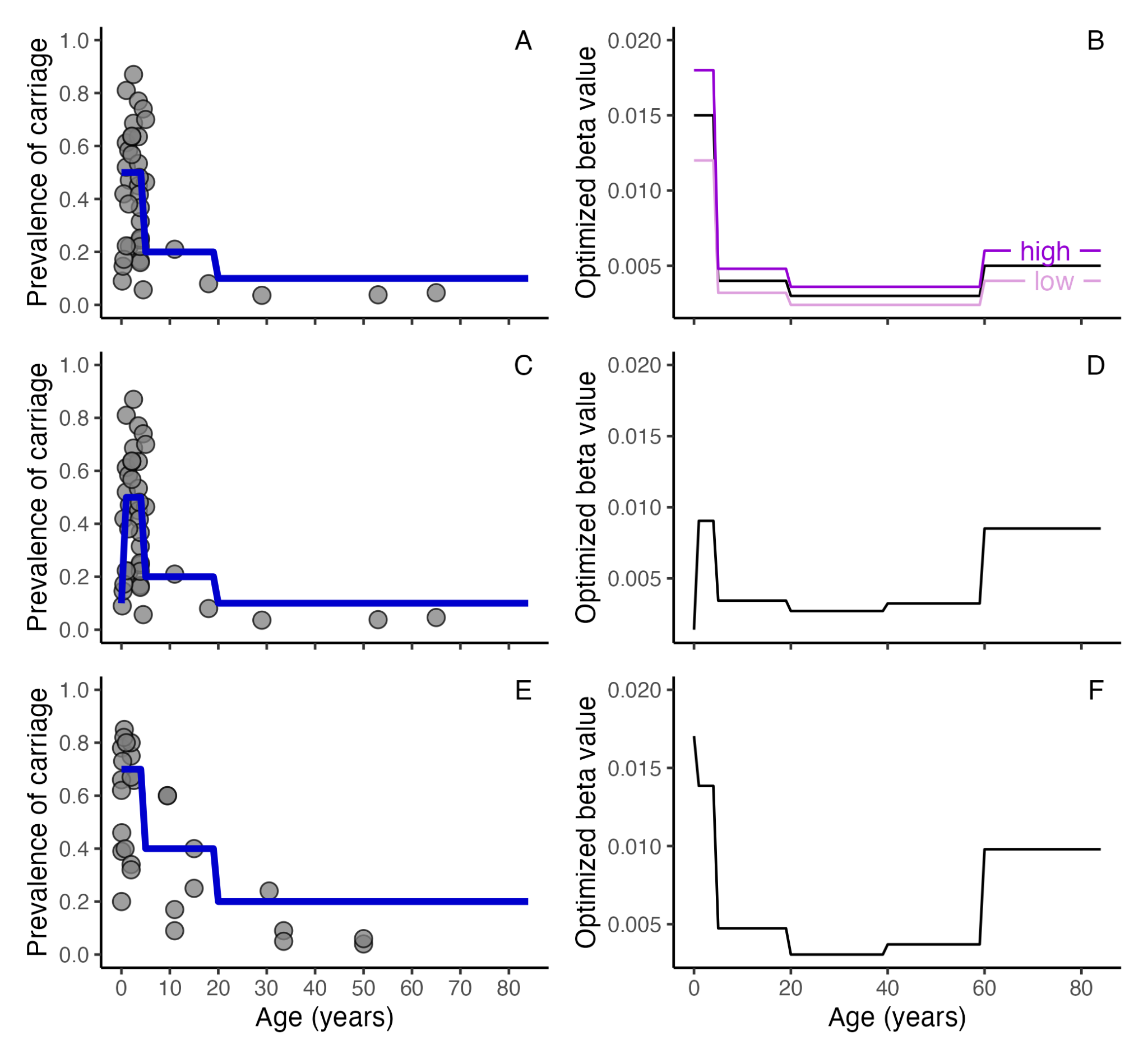


In Supplementary Figure 6 left panels, the points show the observed carriage prevalence by age extracted from published observational studies (Data 2) conducted in high-income countries (HIC) (panels A, C) and low-income countries (LMIC) (panel E); the blue lines mark the assumed initial prevalence of carriers in the main analysis (panel A) and the sensitivity analyses (panels C, E). The right panels show the age-specific susceptibility parameter values used in the main analysis (panel B) and the sensitivity analyses (panels D, F).

​In the main analysis, we assumed the initial overall carriage prevalence to be 50% in age 0-4, 20% in age 5-19, and 10% in age 20-84, based on a review [26], reflecting the carriage prevalence in the settings of high-income countries (panel A). We fixed the age-specific susceptibility parameter $\beta^{(i)}$ for low, medium, and high population susceptibility (panel B) when quantifying the influence of this parameter to time-to-elimination (results in main text Figure 6D).

As sensitivity analyses, we used different susceptibility distributions over age considering two scenarios based on a scoping literature review.

We searched for observational studies on pneumococcal carriage prevalence on PubMed and further identified relevant studies from the references of the initially included studies. We included culture-based studies conducted in the pre-PCV era that reported either overall (including both VT and NVT) or VT carriage prevalence because VT-carriers accounted for most of the carriage in the pre-PCV era. After excluding publications that were based on the same sample, a total of 17 studies were included (HIC: n=11 [14–23,25], LMIC: n=6 [5–8,13,24]). We observed that the carriage prevalence at age 0 was lower than in children aged 1–4, and that carriage prevalence was higher at all ages in LMIC compared with HIC. The income group of countries were based on the World Development Indicators 2008 [27] .

Based on these observations, we considered a lower prevalence of carriers at age 0 (10% instead of 50%) due to the time lag from birth to first pneumococcal acquisition (panel C) and optimized $\beta^{(i)}$ (panel D). The resulted time-to-elimination ranged from 4.2–7.1 years.

We then considered higher carriage prevalences for all ages (70% in age 0–4, 40% in age 5–19, and 20% in age 20–84) to mimic settings with higher pneumococcal burden (panel E), which is commonly observed in low-income countries (LIC) [25,26], and optimized $\beta^{(i)}$ (panel F). The resulted time-to-elimination ranged from 4.4–6.9 years.

**Supplementary Figure 7. Effect of changing vaccine efficacy and coverage on time-to-elimination**


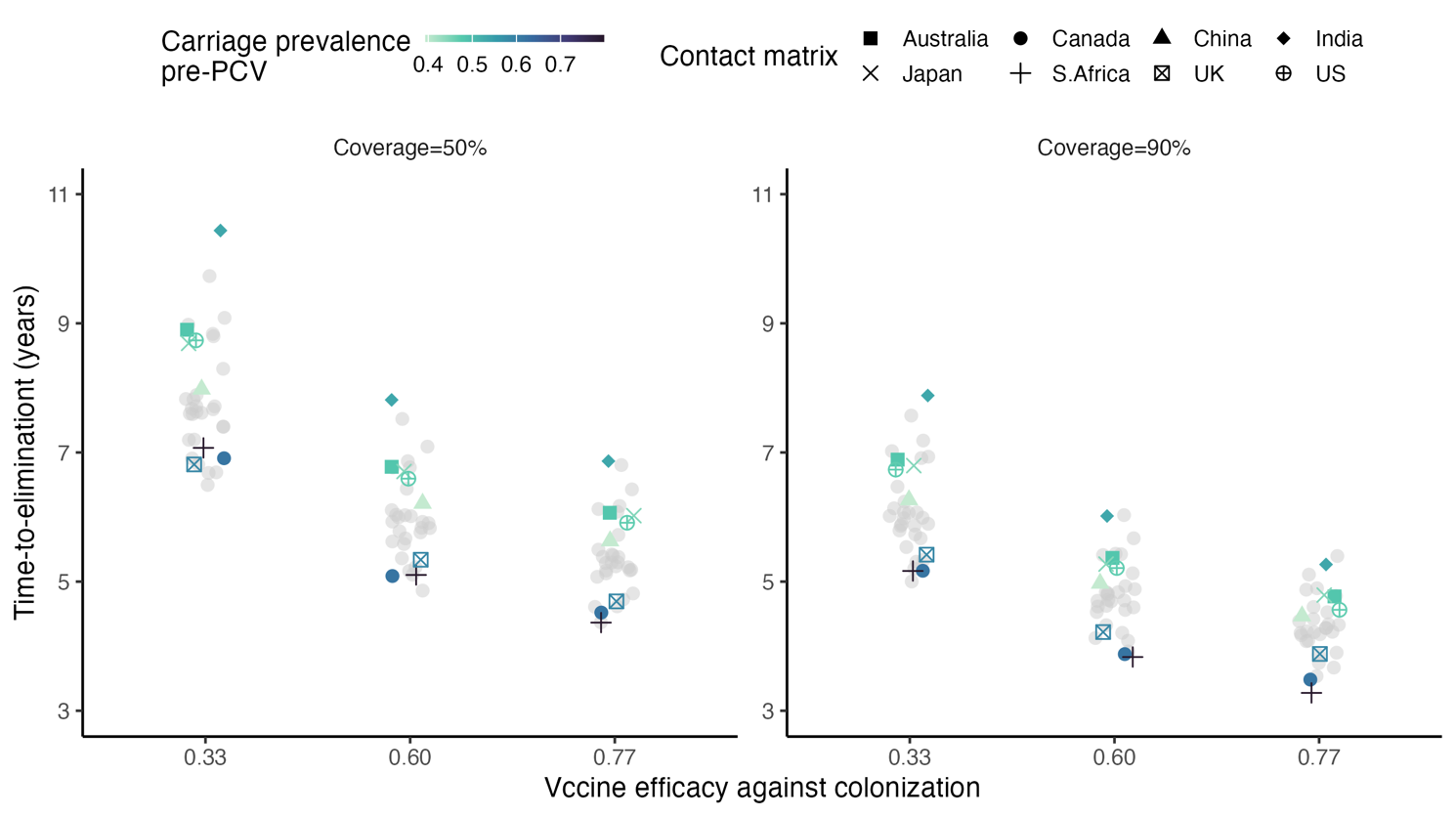


The change in time-to-elimination due to vaccine efficacy was larger when vaccine coverage was lower. At 50% coverage, switching from a less efficacious vaccine (vaccine efficacy=33%) to a highly efficacious vaccine (77%) resulted in 2.5–3.5 years shorter time-to-elimination (left panel). When vaccine coverage reached 90%, using a highly efficacious vaccine led to a 1.7–2.5-year reduction in time-to-elimination compared with a less efficacious vaccine (right panel, same as main text Figure 6A).

**Supplementary Figure 8. Effect of initial proportions of VT, NVT and Co-carriers on time-to-elimination, with changing initial proportion of VT among all colonizing serotypes (**$\boldsymbol{F}$**)**

| $f_{V}\left( 0 \right)$ | $f_{N}\left( 0 \right)$ | $f_{Co}\left( 0 \right)$ | $F$ |
| --- | --- | --- | --- |
| 0.2 | 0.2 | 0.6 | 0.5 |
| 0.4 | 0.2 | 0.4 | 0.6 |
| 0.4 | 0.4 | 0.2 | 0.5 |
| 0.6 | 0.2 | 0.2 | 0.7 |
| 0.6 | 0.4 | 0 | 0.6 |
| 0.8 | 0.2 | 0 | 0.8 |


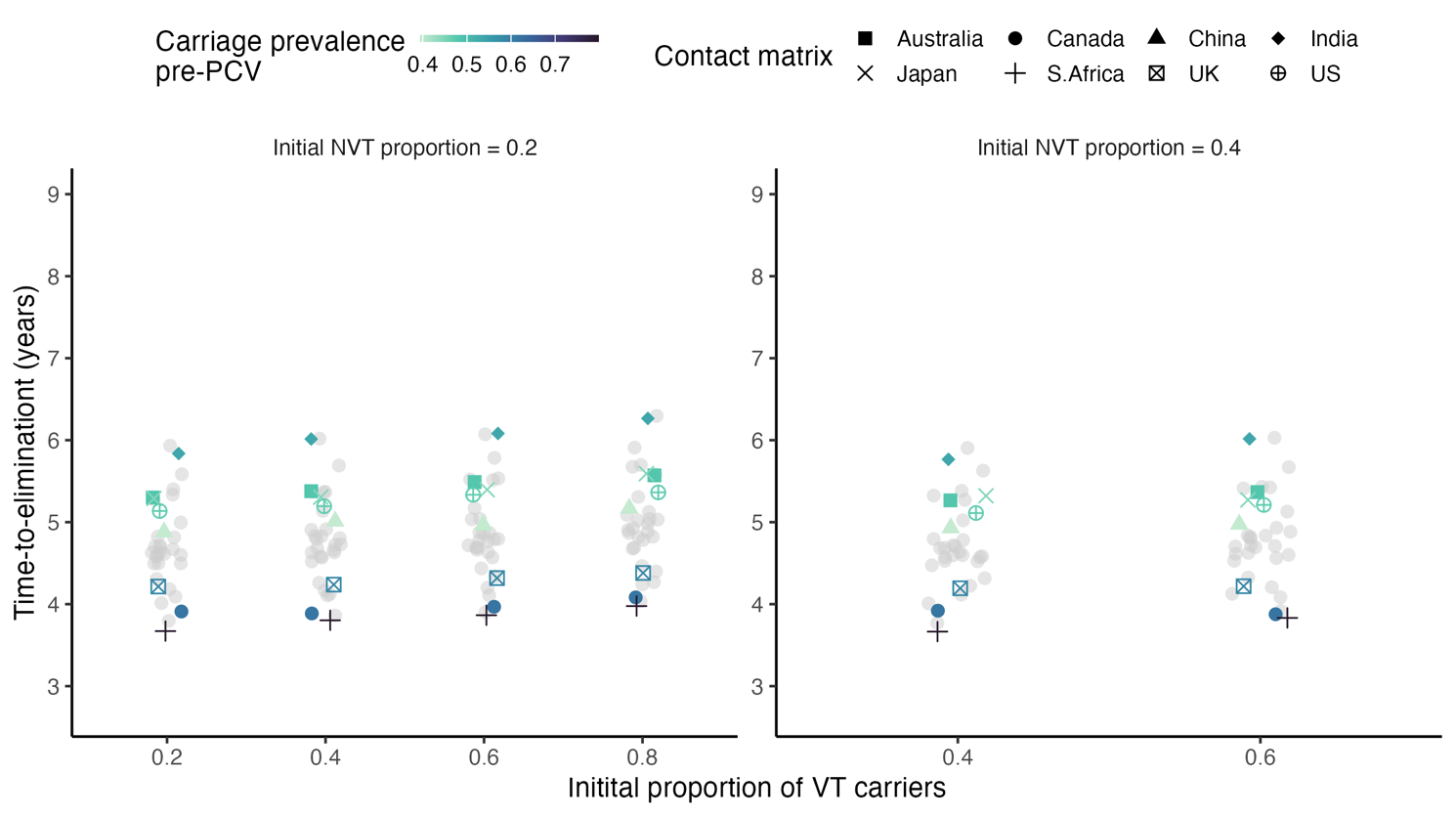


We varied the initial proportion of VT-carriers ($f_{V}\left( 0 \right)$) between 0.2 and 0.8, and the initial proportion of NVT-carriers ($f_{N}\left( 0 \right)$) between 0.2 and 0.4, with the proportion of co-carriers ($f_{Co}\left( 0 \right)$) implicitly determined ($1-f_{V}\left( 0 \right)-f_{N}\left( 0 \right)$). The combinations of initial VT:NVT:Co-carriers ratio were restricted to two conditions: (1) $f_{V}\left( 0 \right)+f_{N}\left( 0 \right)\leq1$ because the sum of proportions cannot exceed one , and (2) $f_{V}\left( 0 \right)\geq f_{N}\left( 0 \right)$ because VT-carriers tended to be more prevalent than NVT carriers in the pre-PCV era (main text Table 1).

When $f_{N}\left( 0 \right)$ was fixed at 0.2, increasing $f_{V}\left( 0 \right)$ from 0.2 to 0.8 resulted in an increase in the initial proportion of VT among all colonizing serotypes ($F$) from 0.5 to 0.8, leading to a slightly longer time-to-elimination (left panel, same as main text Figure 6C).

This effect remained when $f_{N}\left( 0 \right)$ was fixed at 0.4 and $f_{V}\left( 0 \right)$ increased from 0.4 to 0.6, which resulted in an increase in initial $F$ from 0.5 to 0.6.

**Supplementary Figure 9. Effect of initial proportions of VT, NVT and Co-carriers on time-to-elimination, with fixed proportion of VT among all colonizing serotypes (**$\boldsymbol{F}$**)**

| $f_{V}\left( 0 \right)$ | $f_{N}\left( 0 \right)$ | $f_{Co}\left( 0 \right)$ | $F$ |
| --- | --- | --- | --- |
| 0.60 | 0.30 | 0.1 | 0.65 |
| 0.65 | 0.35 | 0 | 0.65 |
| 0.50 | 0.20 | 0.3 | 0.65 |
| 0.40 | 0.10 | 0.5 | 0.65 |


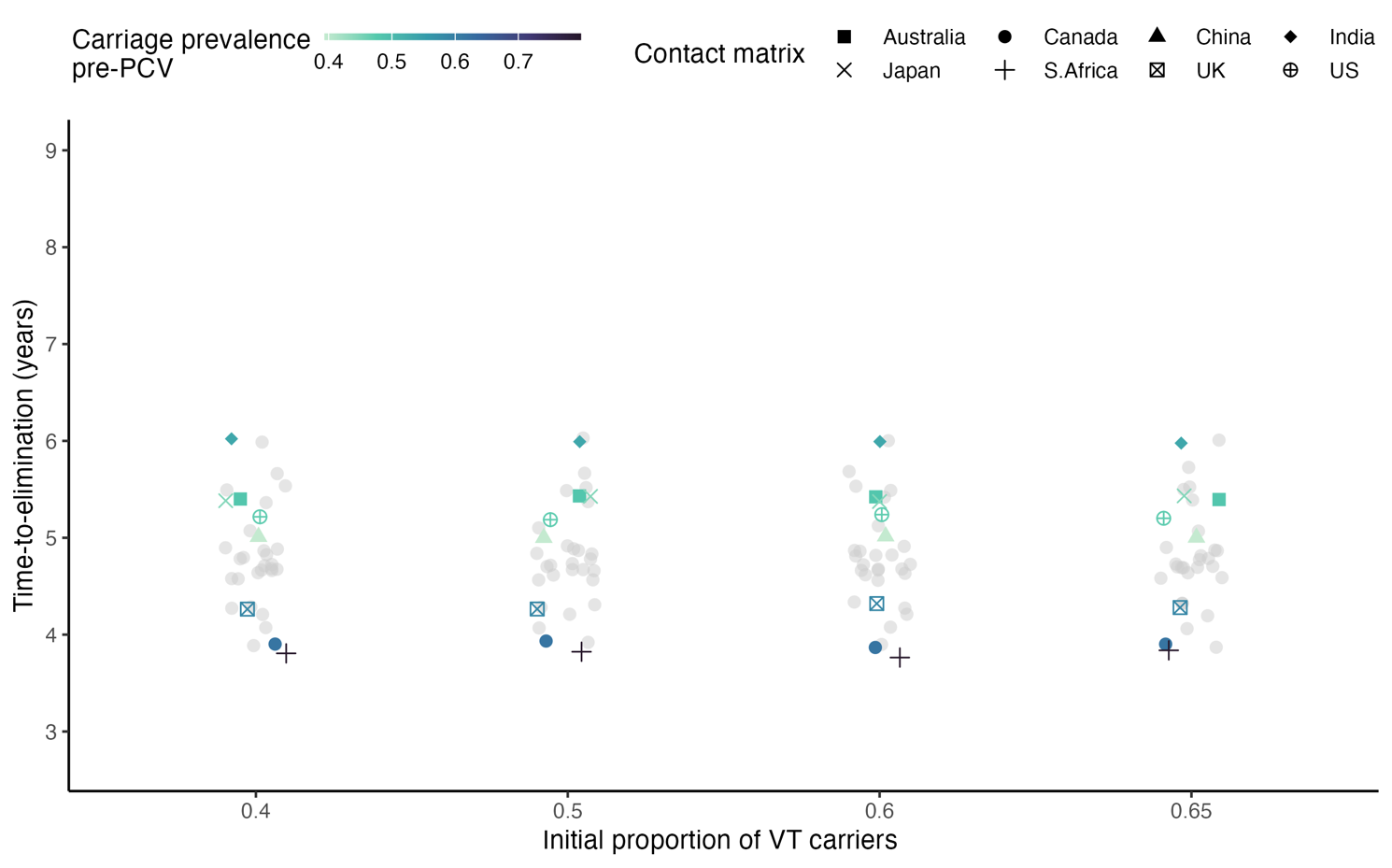


We varied the initial proportion of VT-carriers ($f_{V}\left( 0 \right)$), of NVT-carriers ($f_{N}\left( 0 \right)$), and implicitly, the initial proportion of co-carriers ($1-f_{V}\left( 0 \right)-f_{N}\left( 0 \right)$), such that the initial proportion of VT among all colonizing serotypes ($F$) remained fixed at 0.65. The combinations of initial VT:NVT:Co-carriers ratio were restricted to two conditions: (1) $f_{V}\left( 0 \right)+f_{N}\left( 0 \right)\leq1$ because the sum of proportions cannot exceed one , and (2) $f_{V}\left( 0 \right)\geq f_{N}\left( 0 \right)$ because VT-carriers tended to be more prevalent than NVT carriers in the pre-PCV era (main text Table 1).

With initial $F$ fixed at 0.65, the time-to-elimination remained constant regardless of the initial VT:NVT:Co-carriers ratio.

**Supplementary Figure 10. Effect of competition on time-to-elimination with fixed proportion of VT among all colonizing serotypes (**$\boldsymbol{F}$**)**


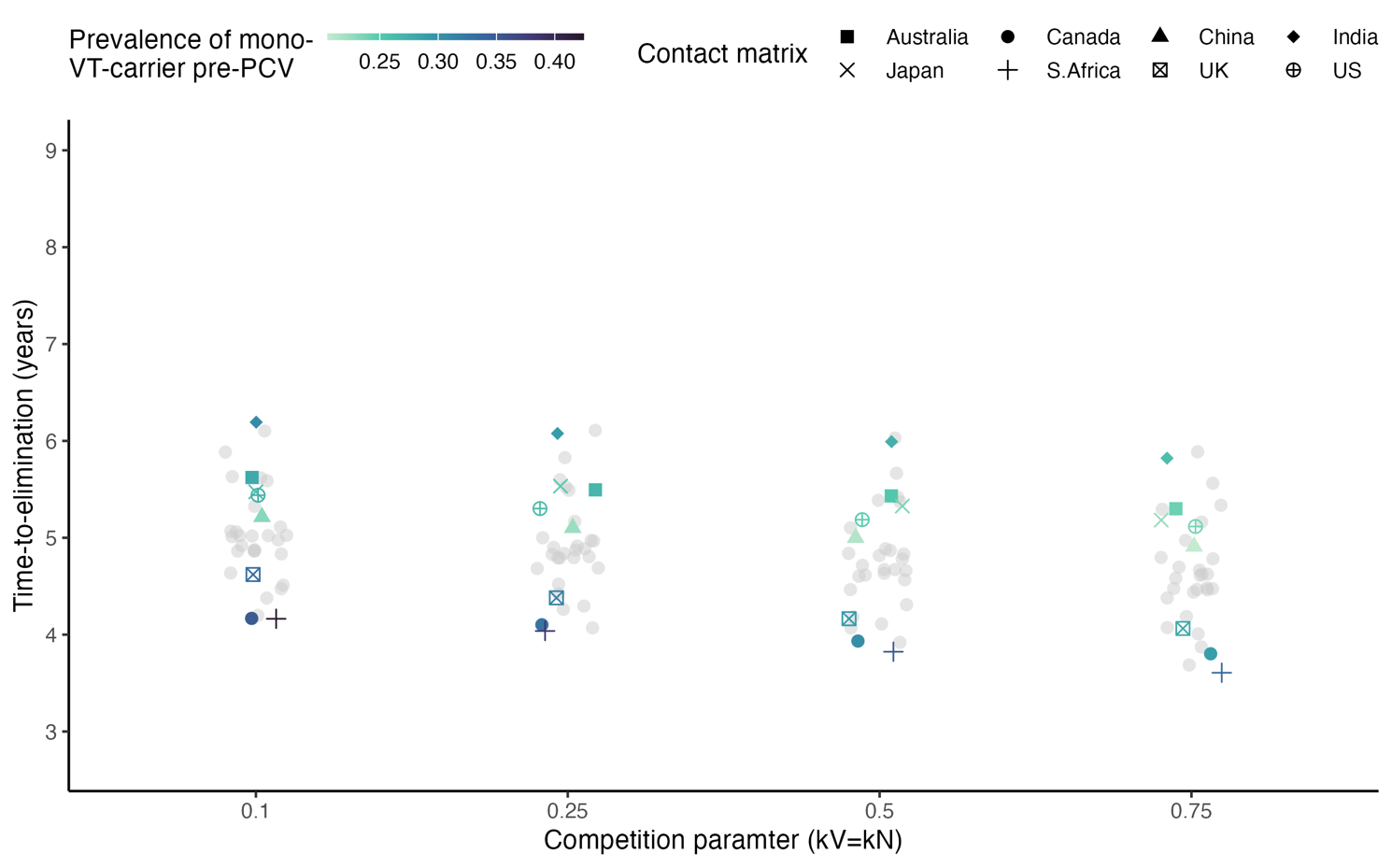


Keeping initial $F$ fixed at 0.65, we tested stronger ($k_{V}$=$k_{N}$=0.1, 0.25) and weaker ($k_{V}$=$k_{N}$=0.75) competitions. We found that time-to-elimination remained similar (time-to-elimination range: 4.2–6.2 years, 4–6.1 years, 3.6­–5.9 years) to that in the main analysis (3.8–6 years).

The competition modelled here referred to the direct competition between VT and NVT in the nasopharynx, such that an existing colonization with VT (or NVT) would reduce colonization with NVT (or VT) by 25% ($k_{V}$=$k_{N}$=0.75), 50% ($k_{V}$=$k_{N}$=0.5), 75% ($k_{V}$=$k_{N}$=0.25), or 90% ($k_{V}$=$k_{N}$=0.1). As competition became stronger, co-carriers became less prevalent while mono-VT carriers and mono-NVT carriers became more prevalent, which led to a longer time for VT to be eliminated.

**Supplementary Figure 11. Simulated time-to-elimination using type I demography vs. empirical demography**

As a sensitivity analysis, we repeated the simulations using country-specific empirical demography from [9] and birth rate from the World Bank Open Data [28]. The simulated time-to-elimination remained similar, with slightly larger deviation seen in countries such as India and South Africa.

**Supplementary Figure 12. Contact features by age group based on type I vs. empirical demography**

**
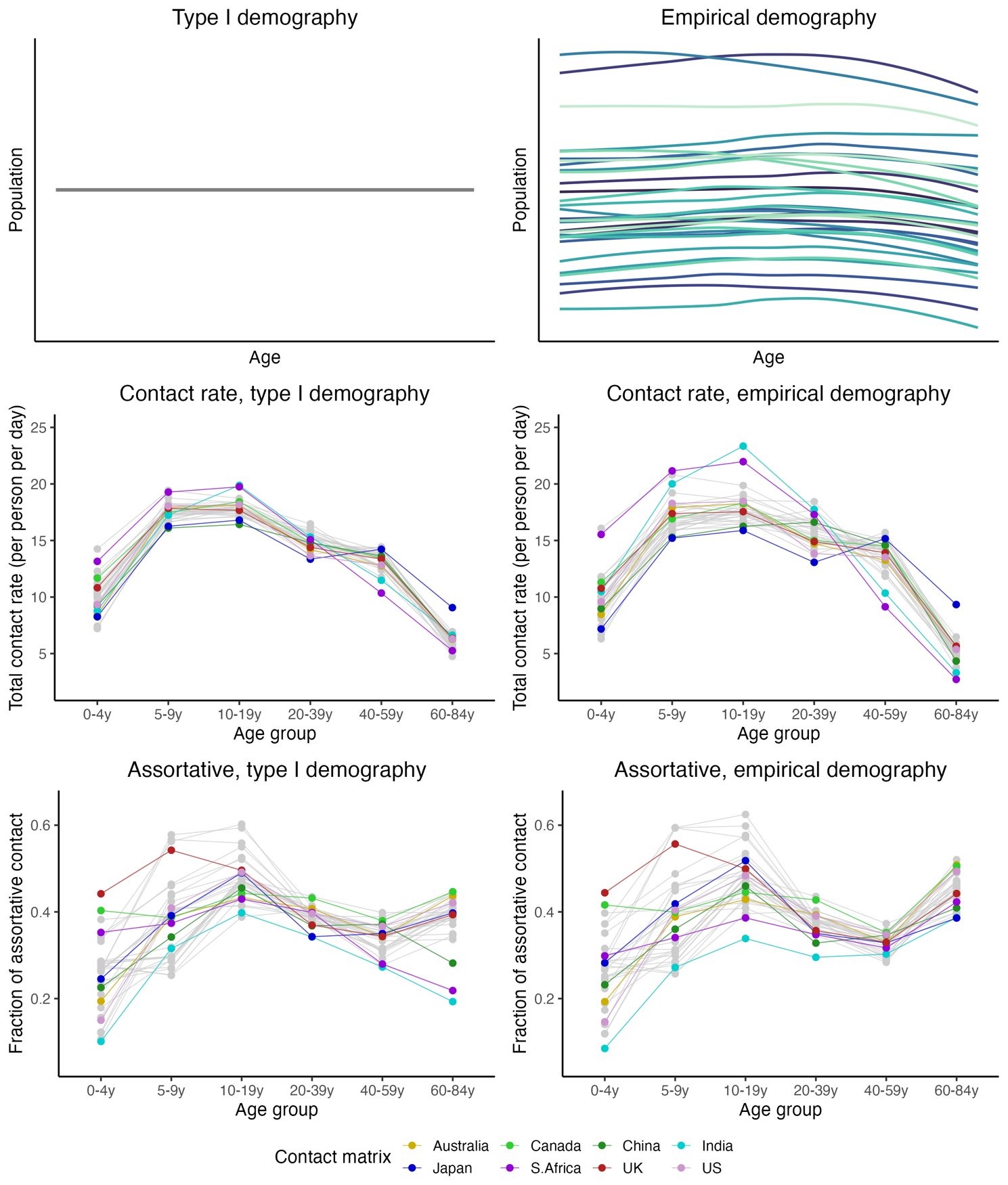
**

Supplementary Figure 12 shows the contact features (contact rate and assortativity) by age group based on type I demography (same for all countries, top left) in the main analysis (middle and bottom left) and those based on empirical demography (from 34 countries, top right) in the sensitivity analyses (middle and bottom right).

**Supplementary Figure 13. Association of features of contact patterns and time-to-elimination simulated with type I demography in all age groups**


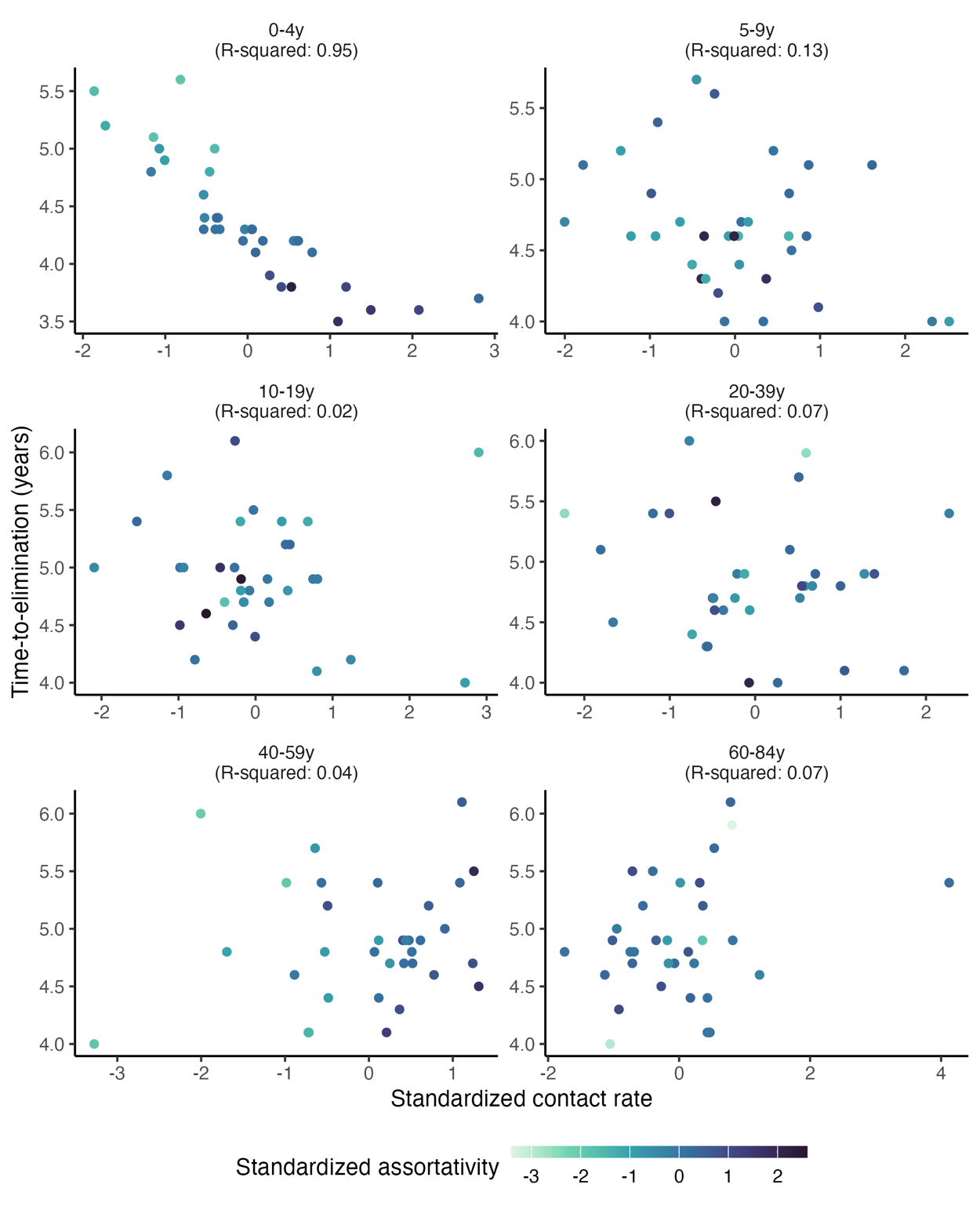


After simulating the transmission using the contact matrices from 34 countries in [9], we explored the relationship between contact features and time-to-elimination in each age group.

The time-to-elimination was measured for each age group. We then calculated the total number of daily contacts in an age group, divided by the age group width, such that this measure would not be inflated in the age groups of wider bands. For example, for age group 0–4y, the total number of daily contacts was divided by 5; for age group 20–39y, the total number of daily contacts was divided by 20. We defined assortativity as the average fraction of contacts from within the age group out of total contact for each age in the age group. We standardized both measures of contact features for easier comparison.

Supplementary Figure 13 shows time-to-elimination’s correlation with standardized contact rate (x-axis) and standardized assortativity (colour scale) for all age groups, revealing a strong trend in children under 5. The R-squared value in each panel indicates the variability in time-to-elimination explained by standardized contact rate and standardized assortativity in a generalized linear model (GLM) for each respective age group.

**Supplementary Figure 14. Association of features of contact patterns and time-to-elimination simulated with empirical demography in all age groups**

**
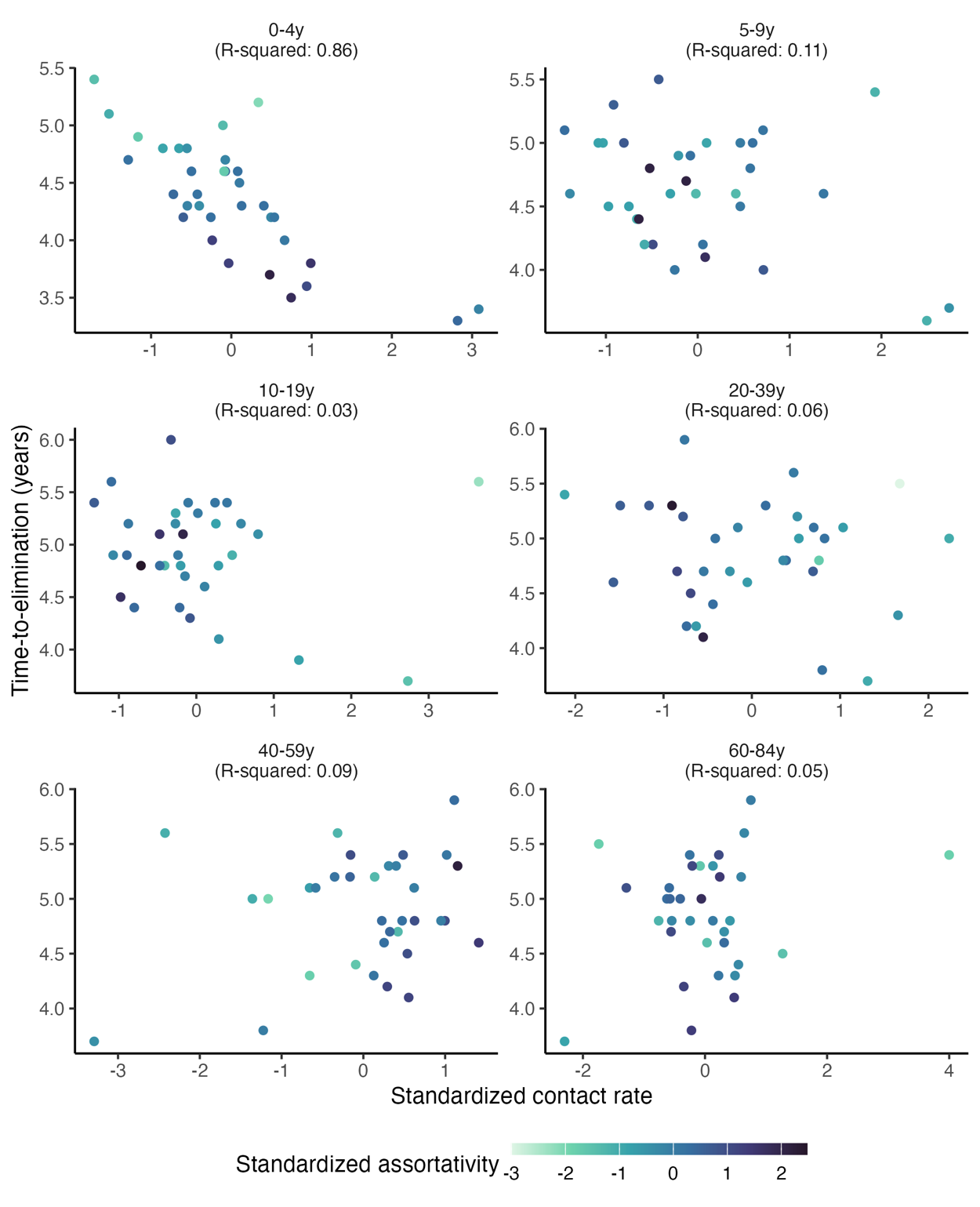
**

Supplementary Figure 14 shows time-to-elimination’s correlation with standardized contact rate (x-axis) and standardized assortativity (colour scale) for all age groups from simulations using empirical demography in a sensitivity analysis. The R-squared value in each panel indicates the variability in time-to-elimination explained by standardized contact rate and standardized assortativity in a generalized linear model (GLM) for each respective age group.

**Supplementary Figure 15. Association of features of contact patterns and time-to-elimination assuming different carriage prevalences**

|  |  | **R-squared in each age group (time-to-elimination ~ contact + assortativity)** | | | | | |
| --- | --- | --- | --- | --- | --- | --- | --- |
| **Analysis** | **Assumed initial prevalence by age** | **0–4y** | **5–9y** | **10–19y** | **20–39y** | **40–59y** | **60–84y** |
| **Main analysis** | $f_{C}^{\left( 0,\ldots,4 \right)}(0)=0.5$  $f_{C}^{\ldots}(0)=0.2$  $f_{C}^{\left( 20,\ldots,59 \right)}\left( 0 \right)=0.1$  $\ldots(0)=0.1$ | 0.95 | 0.13 | 0.02 | 0.07 | 0.04 | 0.07 |
| **Lower prevalence**  **at age 0** | $f_{C}^{\left( 0 \right)}(0)=0.1$  $\ldots(0)=0.\ldots(0)=0.2$  $f_{C}^{\left( 20,\ldots,59 \right)}\left( 0 \right)=\ldots$  $f_{C}^{\left( 60,...,84 \right)}(0)=0.1$ | 0.85 | 0.14 | 0.03 | 0.13 | 0.04 | 0.25 |
| **Higher prevalence at all ages** | $f_{C}^{\left( 0,...,4 \right)}\ldots=0.7$  $f_{C}^{\left( 5,...,19 \right)}(0)=0.4$  $\ldots=0.2$  $f_{C}^{\left( 60,...,84 \right)}(0)=0.2$ | 0.91 | 0.32 | 0.10 | 0.12 | 0.13 | 0.19 |

**
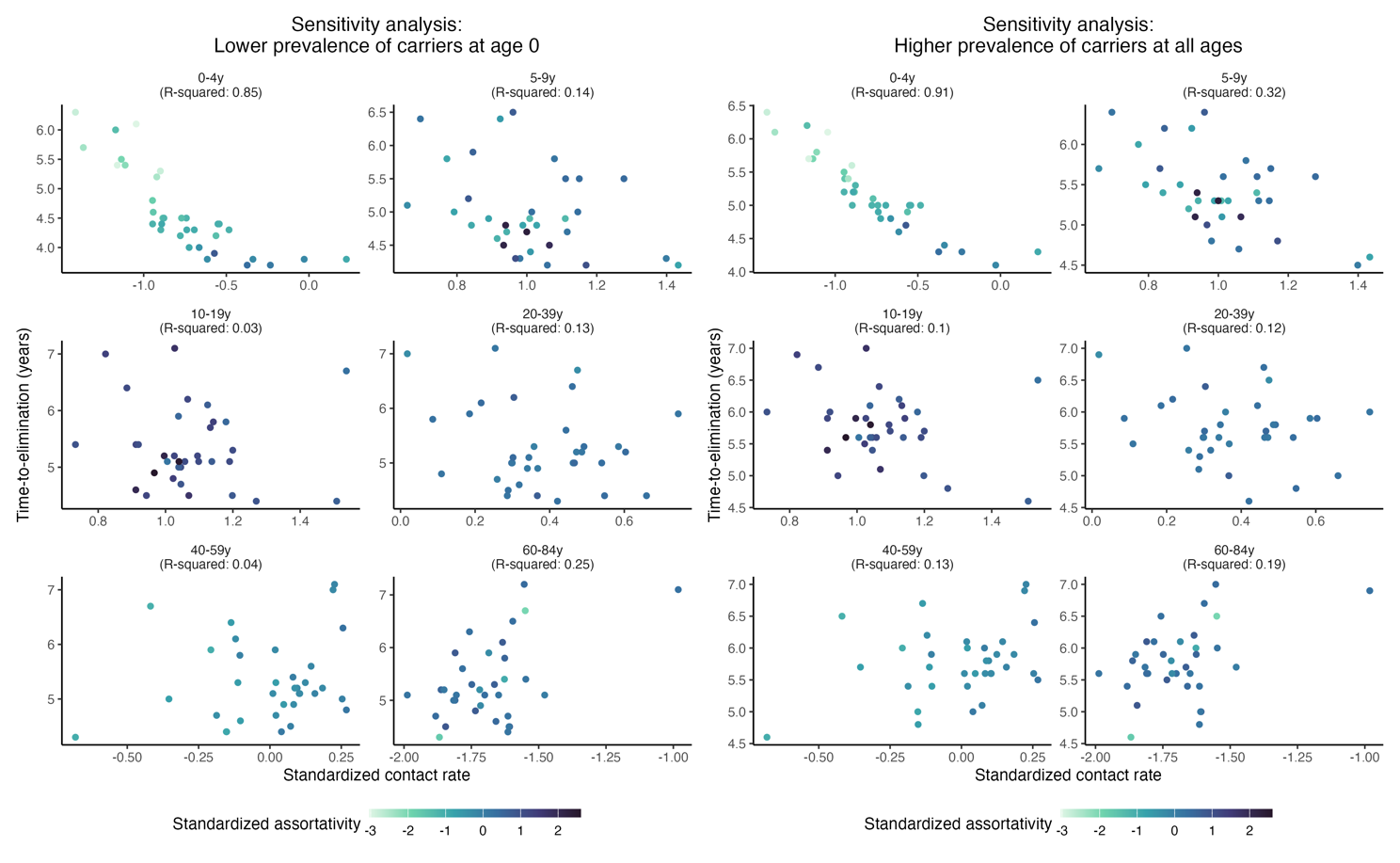
**

Supplementary Figure 15 shows time-to-elimination’s correlation with standardized contact rate (x-axis) and standardized assortativity (colour scale) for all age groups from simulations assuming different carriage prevalences in a sensitivity analysis. The R-squared value in each panel indicates the variability in time-to-elimination explained by standardized contact rate and standardized assortativity in a generalized linear model (GLM) for each respective age group.

**Supplementary Table 2. Out-of-sample prediction using contact rate and assortativity as predictors**

| **Iteration** | **Contact matrices in the test set** | **MAE** | **MRAE** |
| --- | --- | --- | --- |
| 1 | Czech, Finland, Bulgaria, Switzerland | 0.06 | 1.2% |
| 2 | Spain, Netherlands, Bulgaria, United Kingdom | 0.21 | 5.0% |
| 3 | Slovenia, Austria, Ireland, Lithuania | 0.07 | 1.7% |
| 4 | Australia, Norway, Sweden, Austria | 0.09 | 1.9% |
| 5 | Hungary, India, Bulgaria, Ireland | 0.19 | 3.7% |
| 6 | Netherlands, Bulgaria, Austria, India | 0.20 | 4.0% |
| 7 | Cyprus, South Africa, Hungary, United States | 0.06 | 1.5% |
| 8 | United States, Spain, Estonia, Portugal | 0.08 | 1.8% |
| 9 | Finland, Canada, Romania, Italy | 0.08 | 2.2% |
| 10 | France, Canada, Germany, Norway | 0.07 | 1.9% |

To test the predictive performance of the generalized linear model (GLM) using only standardized contact rate and standardized assortativity in children under 5 as predictors, we left 4 randomly selected contact matrices out as the test set and used the remaining 30 contact matrices as the training set. We repeated the procedure for 10 times and reported the mean absolute error (MAE) and mean relative absolute error (MRAE) for each iteration.

We found that a GLM containing only standardized contact rate and standardized assortativity in children under 5 predicted well the time-to-elimination in the test set, with MAE ranging from 0.06 to 0.21 years corresponding to 1.2–5% MRAE in the 10 iterations.

**References**

1. Adler H, German EL, Mitsi E, Nikolaou E, Pojar S, Hales C, et al. Experimental Human Pneumococcal Colonization in Older Adults Is Feasible and Safe, Not Immunogenic. Am J Respir Crit Care Med. 2021;203: 604–613.

2. Ekdahl K, Ahlinder I, Hansson HB, Melander E, Mölstad S, Söderström M, et al. Duration of nasopharyngeal carriage of penicillin-resistant Streptococcus pneumoniae: experiences from the South Swedish Pneumococcal Intervention Project. Clin Infect Dis. 1997;25: 1113–1117.

3. Grivea IN, Priftis KN, Giotas A, Kotzia D, Tsantouli AG, Douros K, et al. Dynamics of pneumococcal carriage among day-care center attendees during the transition from the 7-valent to the higher-valent pneumococcal conjugate vaccines in Greece. Vaccine. 2014;32: 6513–6520.

4. Gritzfeld JF, Cremers AJH, Ferwerda G, Ferreira DM, Kadioglu A, Hermans PWM, et al. Density and duration of experimental human pneumococcal carriage. Clin Microbiol Infect. 2014;20: O1145-51.

5. Hill PC, Cheung YB, Akisanya A, Sankareh K, Lahai G, Greenwood BM, et al. Nasopharyngeal carriage of Streptococcus pneumoniae in Gambian infants: a longitudinal study. Clin Infect Dis. 2008;46: 807–814.

6. Hill PC, Townend J, Antonio M, Akisanya B, Ebruke C, Lahai G, et al. Transmission of Streptococcus pneumoniae in rural Gambian villages: a longitudinal study. Clin Infect Dis. 2010;50: 1468–1476.

7. Jebaraj R, Cherian T, Raghupathy P, Brahmadathan KN, Lalitha MK, Thomas K, et al. Nasopharyngeal colonization of infants in southern India with Streptococcus pneumoniae. Epidemiol Infect. 1999;123: 383–388.

8. Turner P, Turner C, Jankhot A, Helen N, Lee SJ, Day NP, et al. A longitudinal study of Streptococcus pneumoniae carriage in a cohort of infants and their mothers on the Thailand-Myanmar border. PLoS One. 2012;7: e38271.

9. Mistry D, Litvinova M, Pastore Y Piontti A, Chinazzi M, Fumanelli L, Gomes MFC, et al. Inferring high-resolution human mixing patterns for disease modeling. Nat Commun. 2021;12: 323.

10. Arregui S, Aleta A, Sanz J, Moreno Y. Projecting social contact matrices to different demographic structures. PLoS Comput Biol. 2018;14: e1006638.

11. Lipsitch M, Colijn C, Cohen T, Hanage WP, Fraser C. No coexistence for free: neutral null models for multistrain pathogens. Epidemics. 2009;1: 2–13.

12. Demetrius L. Adaptive value, entropy and survivorship curves. Nature. 1978;275: 213–214.

13. Abdullahi O, Karani A, Tigoi CC, Mugo D, Kungu S, Wanjiru E, et al. Rates of acquisition and clearance of pneumococcal serotypes in the nasopharynges of children in Kilifi District, Kenya. J Infect Dis. 2012;206: 1020–1029.

14. Bogaert D, Engelen MN, Timmers-Reker AJ, Elzenaar KP, Peerbooms PG, Coutinho RA, et al. Pneumococcal carriage in children in The Netherlands: a molecular epidemiological study. J Clin Microbiol. 2001;39: 3316–3320.

15. Ekholm A, Jokinen J, Kilpi T. Combining regression and association modelling for longitudinal data on bacterial carriage. Stat Med. 2002;21: 773–791.

16. Gray BM, Converse GM 3rd, Huhta N, Johnston RB Jr, Pichichero ME, Schiffman G, et al. Epidemiologic studies of Streptococcus pneumoniae in infants: antibody response to nasopharyngeal carriage of types 3, 19, and 23. J Infect Dis. 1981;144: 312–318.

17. Grivea IN, Panagiotou M, Tsantouli AG, Syrogiannopoulos GA. Impact of heptavalent pneumococcal conjugate vaccine on nasopharyngeal carriage of penicillin-resistant Streptococcus pneumoniae among day-care center attendees in central Greece. Pediatr Inf

ct Dis J. 2008;27: 519–525.

18. Guillemot D, Carbon C, Balkau B, Geslin P, Lecoeur H, Vauzelle-Kervroëdan F, et al. Low dosage and long treatment duration of beta-lactam: risk factors for carriage of penicillin-resistant Streptococcus pneumoniae. JAMA. 1998;279: 365–370.

19. Hussain M, Melegaro A, Pebody RG, George R, Edmunds WJ, Talukdar R, et al. A longitudinal household study of Streptococcus pneumoniae nasopharyngeal carriage in a UK setting. Epidemiol Infect. 2005;133: 891–898.

20. Leino T, Hoti F, Syrjänen R, Tanskanen A, Auranen K. Clustering of serotypes in a longitudinal study of Streptococcus pneumoniae carriage in three day care centres. BMC Infect Dis. 2008;8: 173.

21. Regev-Yochay G, Raz M, Dagan R, Porat N, Shainberg B, Pinco E, et al. Nasopharyngeal carriage of Streptococcus pneumoniae by adults and children in community and family settings. Clin Infect Dis. 2004;38: 632–639.

22. Sá-Leão R, Nunes S, Brito-Avô A, Alves CR, Carriço JA, Saldanha J, et al. High rates of transmission of and colonization by Streptococcus pneumoniae and Haemophilus influenzae within a day care center revealed in a longitudinal study. J Clin Microbiol. 2008;46: 225–234.

23. Vestrheim DF, Høiby EA, Aaberge IS, Caugant DA. Phenotypic and genotypic characterization of Streptococcus pneumoniae strains colonizing children attending day-care centers in Norway. J Clin Microbiol. 2008;46: 2508–2518.

24. Hammitt LL, Akech DO, Morpeth SC, Karani A, Kihuha N, Nyongesa S, et al. Population effect of 10-valent pneumococcal conjugate vaccine on nasopharyngeal carriage of Streptococcus pneumoniae and non-typeable Haemophilus influenzae in Kilifi, Kenya: findings from cross-sectional carriage studies. Lancet Glob Health. 2014;2: e397-405.

25. O’Brien KL, Millar EV, Zell ER, Bronsdon M, Weatherholtz R, Reid R, et al. Effect of pneumococcal conjugate vaccine on nasopharyngeal colonization among immunized and unimmunized children in a community-randomized trial. J Infect Dis. 2007;196: 1211–1220.

26. Clifford S, Knoll MD, O’Brien KL, Pollington TM, Moodley R, Prieto-Merino D, et al. Global landscape of*Streptococcus pneumoniae*serotypes colonising healthy individuals worldwide before vaccine introduction; a systematic review and meta-analysis. bioRxiv. 2023. doi:10.1101/2023.03.09.23287027

27. Cieslikowski, David. World development indicators 2008. In: World Bank [Internet]. Cieslikowski, David,; [cited 27 Jun 2024]. Available: http://documents.worldbank.org/curated/en/587251468176971009/World-development-indicators-2008

28. World Bank Open Data. In: World Bank Open Data [Internet]. [cited 6 Jul 2025]. Available: https://data.worldbank.org
